# PQBP1 couples HIV-1 capsid recognition to cGAS recruitment through conformational remodeling

**DOI:** 10.64898/2026.07.07.737097

**Published:** 2026-07-31

**Authors:** Dale S Allen, Asit Manna, Christian Michel Beusch, Tianhao Zhang, Barbie K Ganser-Pornillos, Juliana Piacentini, Seolhwa Oh, Savni Prabhu, Ayush Mehta, David Gordon, Owen Pornillos, Sunnie M Yoh, Sumit K Chanda

## Abstract

Pattern recognition receptors (PRRs) must selectively engage pathogen-derived signals to potentiate inflammation and antimicrobial responses. Polyglutamine-binding protein 1 (PQBP1) acts upstream of cyclic GMP-AMP synthase (cGAS) during HIV-1 infection through the recognition of viral capsid. However, the mechanism by which capsid binding enables cGAS recruitment remains unclear. As an intrinsically disordered protein, PQBP1 samples an ensemble of conformational states whose distribution depends on ligand engagement. Here we show that capsid engagement shifts this population toward a conformation competent for cGAS binding. Capsid binding at the N-terminus of PQBP1 redistributes conformational sampling within the WW domain and distal polar-rich domain (PRD). Alanine substitutions within these capsid-responsive regions retain capsid binding yet disrupt infection-dependent cGAS recruitment, indicating that capsid binding and cGAS recruitment depend on distinct regions of PQBP1. Together, these findings define a mechanism by which HIV-1 capsid engagement remodels PQBP1 into a cGAS-competent state, coupling capsid recognition to innate immune activation.

**IMPORTANCE:**

Polyglutamine-binding protein 1 (PQBP1) initiates innate immune detection of HIV-1 by recognizing the incoming viral capsid. We show that capsid engagement reshapes PQBP1 conformational dynamics, and we identify distal regions required for infection-dependent cGAS association. These findings provide a mechanistic framework for how pathogen recognition is coupled to downstream innate immune activation.

## Full Text Availability

The license terms selected by the author(s) for this preprint version do not permit archiving in PMC. The full text is available from the preprint server.

